# Effectiveness of HEPA/Carbon Filter Air Purifier in Reducing Indoor NO_2_ and PM_2.5_ in Homes with Gas Stove Use in Lowell, Massachusetts

**DOI:** 10.3390/toxics13121030

**Published:** 2025-11-28

**Authors:** Khafayat Kadiri, David Turcotte, Rebecca Gore, Anila Bello, Serena Rajabiun, Karyn Heavner, Susan R. Woskie

**Affiliations:** 1 Department of Public Health, University of Massachusetts Lowell, Lowell, MA 01854, USA; anila_bello@uml.edu (A.B.); serena_rajabiun@uml.edu (S.R.); karyn_heavner@uml.edu (K.H.); susan_woskie@uml.edu (S.R.W.); 2 Center for Community Research and Engagement, University of Massachusetts Lowell, Lowell, MA 01854, USA; david_turcotte@uml.edu; 3 Department of Biomedical Engineering, University of Massachusetts Lowell, Lowell, MA 01854, USA; rebecca_gore@uml.edu

**Keywords:** nitrogen dioxide (NO_2_), particulate matter (PM), HEPA/carbon filters, gas stoves

## Abstract

Nitrogen dioxide (NO_2_) and particulate matter of 2.5 microns (PM_2.5_) impact health outcomes. This study utilized a pre- to post-test study design to evaluate the impact of air purifiers fitted with a high-efficiency particulate air (HEPA) and carbon filters in reducing indoor NO_2_ and PM_2.5_. Sixty-seven low-income homes in Lowell, Massachusetts, were included in this study. Home visits were conducted every four months for 12 months. At each visit, we conducted environmental sampling, measuring indoor NO_2_, PM_2.5_, stove use, temperature, and humidity over 5–7 days. We collected environmental exposure data using questionnaires. Air purifiers were introduced after the 4th month. Linear mixed models were used to predict changes in NO_2_ and PM_2.5_, with independent predictors as fixed effects and homes as random effects. The geometric mean (GM) for NO_2_ decreased by 36% from 20.16 to 12.79 ppb (*p* < 0.001). GM for PM_2.5_ decreased by 45% from 17.12 to 9.16 µg/m^3^ (*p* < 0.001). We found that an increase in air purifier use resulted in a significant decrease in NO_2_ and PM_2.5_, and an increase in stove usage increased NO_2_. HEPA/carbon filters have the potential to improve indoor air quality by reducing NO_2_ and PM_2.5,_ enabling the tailoring of interventions to mitigate these air pollutants.

## 1. Introduction

Approximately thirty-eight percent (38%) of the United States (U.S) population uses gas stoves [1]. Gas stoves utilize natural gas (methane) or Liquefied Petroleum Gas (LPG) (typically a mix of propane and butane), commonly referred to as propane, as their primary carbon source. The combustion of these gases produces pollutants, including nitrogen dioxide (NO_2_) and particulate matter with a diameter of 2.5 microns (PM_2.5_) [2]. Gas stoves present a heightened health risk from the close proximity of individuals to concentrated emissions during cooking activities [3,4,5]. Indoor combustion from gas stoves has been identified as an important source of indoor NO_2_ and PM_2.5_ [2,6,7,8]. During cooking, peaks of NO_2_ are generated that may exceed 100 parts per billion (ppb), which is the outdoor short-term 1 h National Ambient Air Quality Standard (NAAQS), within a couple of minutes [3,4,5]. A study by Paulin et al. (2017) found that 37% and 12% of NO_2_ sampling days conducted in homes in Baltimore City, Maryland, had 24 h indoor concentrations surpassing the U.S. Environmental Protection Agency (EPA) annual mean outdoor standard of 53 ppb and 1 h outdoor limit of 100 ppb, respectively [5]. Personal exposure to NO_2_ is higher for individuals residing in homes with gas stoves compared to those in homes with electric stoves [9]. Homes without exhaust hoods have been observed to have NO_2_ concentrations exceeding the outdoor standard [10]. Low ventilation and NO_2_ emissions from gas stoves result in a high indoor concentration of NO_2_ during colder months [11].

A study conducted in Albuquerque, New Mexico, to evaluate indoor NO_2_ observed activity rooms found that NO_2_ levels were between 7 and 168.7 ppb in homes with gas stoves and between 2 and 22.7 ppb in homes with electric stoves [12]. This indicates that in these homes with gas stoves, NO_2_ levels exceed EPA-recommended outdoor levels of 100 ppb (daily maximum of 1 h) and 53 ppb (annual average) [13]. A Simulation study in 1000 homes in Boston, Massachusetts, observed cooking as the most significant contributor to modeled indoor concentrations of PM_2.5_, with a mean contribution of 25.6 μg/m^3^ [14]. A study by Li et al. (2017) conducted in Northwest China observed average kitchen PM_2.5_ concentrations in homes with gas stoves at 92, 115, 131, and 135 μg/m^3^ when the daily cooking frequency was 0, 1, 2, and 3, respectively [15]. Li et al. (2017) also observed average PM_2.5_ concentrations of 114 ± 39 µg/m^3^ and 139 ± 99 µg/m^3^ in the kitchen and bedroom, respectively [15]. This indicates that in these homes with gas stoves, PM_2.5_ levels exceed the EPA-recommended outdoor levels of 35 μg/m^3^ (daily maximum of 1 h) and 9 μg/m^3^ (annual average) [13].

Short- and long-term exposure to NO_2_ and PM_2.5_ influences many adverse health effects. Short-term exposure to NO_2_ and PM_2.5_ occurs over hours to a few days, often based on 24 h averages. Short-term exposure to high levels of NO_2_ and PM_2.5_ can trigger acute health effects, including respiratory issues, asthma exacerbations, and increased hospital admissions [13]. Long-term exposure to NO_2_ and PM_2.5_ occurs over months to years and is commonly assessed using annual averages. Long-term exposure to NO_2_ and PM_2.5_ can lead to chronic health problems, such as increased risks of lung diseases, cardiovascular diseases, reduced lung function, and premature death [13]. A multicenter cohort study found that using gas stoves in the home was associated with the risk of certain respiratory symptoms, compared to using electric cookers [16]. Epidemiologic studies have observed associations between exposure to NO_2_ and increased asthma symptoms, including chest tightness, shortness of breath, wheeze, and cough; an increase in rescue inhaler use [17]. Currently, there are no indoor standards for indoor levels of NO_2_ and PM_2.5_ [13] that will protect individuals from the potential harm from these gas stove emissions. This emphasizes the need to investigate ways to reduce these pollutants in the indoor environment. The American Public Health Association (APHA) released a policy statement, highlighting the need to curb gas stove emissions, particularly NO_2_, and indicated the need for research to identify effective interventions and the benefits of interventions [18].

Intervention strategies to reduce NO_2_ and PM_2.5_ exposure have been conducted in randomized trials by replacing gas stoves with electric stoves, installing ventilation hoods, and using portable air purifiers to reduce NO_2_ and PM_2.5_ concentrations in indoor environments [3,19]. Due to the financial cost, replacing gas stoves with electric stoves and installing ventilation that exhausts outside are less likely to be attained in low-income households and transitional living [20]. Recent studies on high-efficiency particulate air (HEPA) filter intervention have shown that reducing NO_2_ and PM_2.5_ levels improves asthma control in children and enhances respiratory function due to their ability to capture airborne particles [21,22,23,24,25,26,27]. HEPA filters effectively reduce at least 99.95% of particles with a diameter of 0.3 μm and larger [28]. A review by Cheek et al. (2021) found that using a portable air purifier resulted in short-term reductions in PM_2.5_ levels within the indoor environment [29]. Most HEPA filter intervention studies have focused on the short-term impact on NO_2_ and PM_2.5_ indoor concentrations.

There is promising evidence that the use of air purifiers with HEPA/carbon filters can effectively reduce NO_2_ and PM_2.5_. The use of air cleaners with HEPA and carbon filters in the homes of former smokers with Chronic Obstructive Pulmonary Disease (COPD) was associated with a 61% greater reduction in indoor PM_2.5_ concentrations and a 24% reduction in NO_2_ concentrations at 6 months compared with homes with sham air cleaners [30]. A study by Paulin et al. (2014) found that introducing air purifiers with HEPA filters reduced the median indoor NO_2_ level by 27% after 1 week and by 19% after 3 months [19]. Charcoal absorbent filters, which are also known as activated carbon filters, have been observed to reduce vehicular NO_2_ through absorption [31]. Activated carbon is a porous material with a vast surface area. This surface area is significantly increased when activated, making it an excellent adsorbent. As air passes through the charcoal sorbent filter, NO_2_ molecules come into contact with the surface of the activated charcoal. The attractive forces between the NO_2_ molecules and the carbon atoms cause NO_2_ to absorb onto the surface. The NO_2_ remains trapped in the activated carbon filter, effectively removing it from the air [31].

The current gaps and limitations in the existing literature require further studies on the long-term duration of filtration use, paired with personal exposure monitoring in the general and vulnerable populations [29]. There has been no published research examining the impact of air purifiers fitted with HEPA/carbon filters on indoor NO_2_ and PM_2.5_ in low-income housing. This study was designed to evaluate the effectiveness of air purifiers fitted with HEPA/carbon filters on indoor NO_2_ and PM_2.5_ concentrations in low-income homes with gas stoves.

## 2. Materials and Methods

The data utilized in this study were obtained from 67 residential apartments as part of an intervention study focused on reducing indoor NO_2_ and PM_2.5_ in homes of older adults diagnosed with asthma residing in low-income housing in Lowell, Massachusetts. The study was conducted between December 2020 and October 2023. Study participants were individuals aged ≥ 55 years, who used a gas stove, resided in Lowell, MA, and had no prior interventions to reduce NO_2_ and PM_2.5_. We excluded individuals with a history of respiratory failure, life-threatening asthma, current residents of a long-term care facility, mental incoherency, and/or participating in other interventional studies. We contacted and recruited these participants within Lowell senior public and private subsidized housing developments with phone calls, direct mailings, and visits to their residences. Human subjects approval was received from the University of Massachusetts Lowell Institutional Review Board (IRB) (Approval number 17-010-TUR-XPD), which initially required a written informed consent form in English, Spanish, or Khmer. However, due to the Coronavirus Disease (COVID-19), we obtained verbal consent to reduce the risk of exposure to COVID-19. We conducted environmental sampling for NO_2_ and PM_2.5_, self-reported environmental triggers, and conducted a visual home assessment for environmental triggers at visits 3 and 4. Other protocol changes due to COVID-19 included community health workers (CHWs) collecting questionnaire data over the phone and delaying visual assessment using a checklist until the third visit (see data collection/intervention schedule below, Table 1). Quantitative and qualitative data were collected and managed using the Research Electronic Data Capture (REDCap) version 15.0.3 electronic data capture tool hosted at the University of Massachusetts Chan Medical School. REDCap is a secure, web-based software that supports data collection for research studies [32,33].

### 2.1. Indoor Environmental Sampling

Our primary outcomes were indoor NO_2_ and PM_2.5_. Environmental sampling was conducted in 67 homes for 5–7 consecutive days, simultaneously measuring NO_2_, PM_2.5_, temperature, humidity, and fractional stove use during four different home visits (Table 1). Environmental sampling was conducted four times (home visits 1, 2, 3, and 4) for each participant between December 2020 and October 2023. NO_2_ sampling was accomplished with a NO_2_ Ogawa sampler (Ogawa and Co, USA Inc. Pompano Beach, FL, USA). The NO_2_ sampler was positioned close to a temperature logger (TRIX-8 multi-use) (manufactured by Logtag North America Inc., Lafayette, NJ, USA [34]), which measured humidity and temperature. NO_2_ samples were shipped by colorimetry to RTI International (Durham, NC, USA) for analysis, which reported a blank-adjusted concentration in ppb using the mean humidity and temperature data during the sampling period [35].

PM_2.5_ concentrations were measured with the Dylos 1700-PM (Dylos Corporation, Riverside, CA, USA) [36,37,38], it is a direct reading optical particle counter. Data from the Dylos was downloaded using the Dylos logger software, and the mean and maximum PM_2.5_ concentrations were recorded. All the sampling devices except the ibutton were placed on a table in the living area as close as possible to the kitchen, where the gas stove was located. Stove usage was measured with an iButton semiconductor temperature sensor (DS1921G-F5, Analog Devices, Inc., Wilmington, MA, USA). This device has been previously used in studies to measure stove temperature changes [39]. Stove use was determined by the fraction of 6 min intervals during which the temperature on the stove’s surface exceeded the background level. Given the potential variability of background temperature in each house, the cut point was determined based on a 2 °C increase from the average temperature from the Logtag data for each home, or 25 °C if the average temperature was <25 °C. Kadiri et al. (2024) provided a more thorough description of the placement of the sampling devices and methods [35].

### 2.2. Interventions

The intervention in this study was a Healthmate air purifier, which consisted of HEPA and carbon filters, to reduce NO_2_ and PM_2.5_. At the end of the second visit, two units were provided in each home, one in the living room and the other in the bedroom. Participants were encouraged to use these air purifiers often and set them to the highest air filtration volume (fan speed) during the study. The participants were educated on how to clean the body of the air purifier to prevent excessive dust accumulation. And the air purifier had a 5-year warranty. The air purifier has been used in previous studies [19,40]. In this study, we installed timers in the air purifiers (L4 Series, ENM Company, Chicago, IL, USA), which allowed for the quantification of air purifier usage during the intervention period, although not the fan speed setting. The time of air purifier usage was determined as the percentage of time used from drop-off to the next home visit. The intervention introduction timeline is detailed in Table 1.

### 2.3. Statistical Analysis

To analyze the impact of the air purifiers, we utilized linear mixed effects models (LMM) to predict continuous outcomes. LMM allowed for the accounting of repeated measurements within households and handled unbalanced datasets. All models included fixed effects (air purifier usage) and a random effect for households to capture variability within households. Continuous outcomes include the natural log of NO_2_ and PM_2.5_ concentrations. Due to the skewed nature of environmental measurements, NO_2_ and PM_2.5_ were log-transformed. The regression analyses also controlled for other independent predictors beyond the evaluated intervention. These included stove usage, season, temperature, humidity, and home characteristics were explained in detail in an earlier study [35]. A significance level (α) of 0.05 was used for all statistical tests. All *p*-values were two-sided to account for the possibility of effects in either direction. Simple linear mixed models were initially investigated to evaluate other fixed predictors for NO_2_ and PM_2.5_, and those statistically significant were included in multiple predictor fixed effect models. For the multiple LMMs, model selection was performed using stepwise selection based on Akaike Information Criterion (AIC) and Bayesian Information Criterion (BIC) values. The primary outcome was the estimated effect of air purifiers on indoor NO_2_ and PM_2.5_, with coefficients (β) indicating the expected change in the natural log of the concentrations of these pollutants. We estimated the percentage change by exponentiating the unadjusted coefficients (β) of NO_2_ and PM_2.5_ in relation to air purifier usage (percent change = 100 × (e^β^ − 1)). The data used for this analysis consisted of pre-intervention data (from visits 1 and 2) and post-intervention data (from visits 3 and 4). All analyses were performed using SAS version 9.4 software (SAS Institute Inc., Cary, NC, USA).

## 3. Results

**Figure 1:**
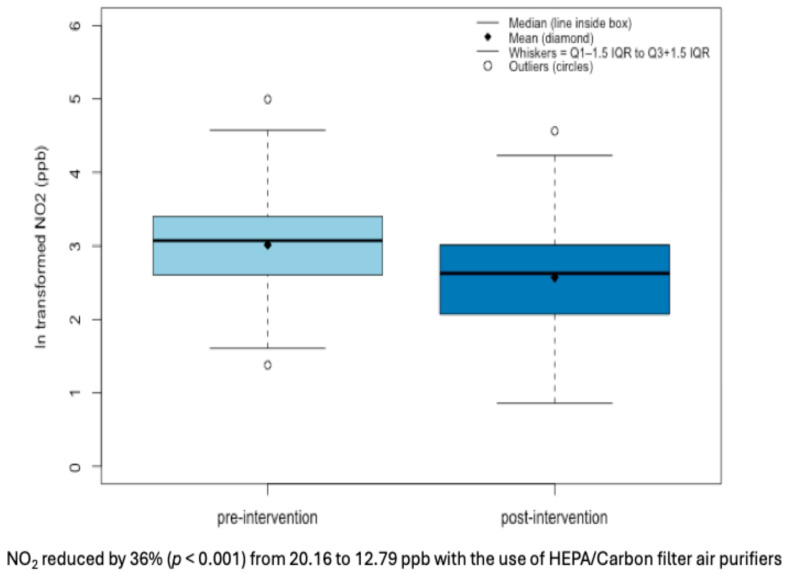
Box plots of indoor ln-transformed NO_2_ levels by intervention timeline.

**Figure 2:**
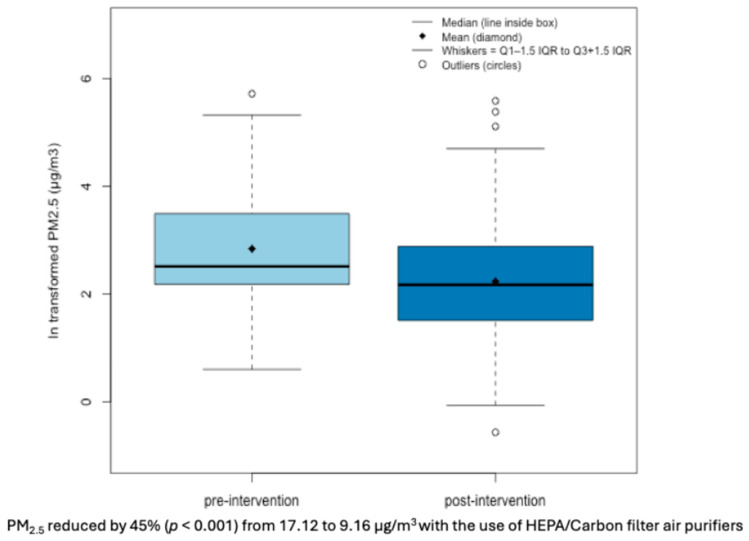
Box plots of indoor ln-transformed PM_2.5_ levels by intervention timeline.

We observed an indoor geometric mean (GM) of NO_2_ to be 20.16 and 12.79 ppb at the pre- and post-intervention periods, respectively, as shown in Figure 1. We observed an indoor GM of PM_2.5_ to be 17.12 and 9.16 µg/m^3^ at pre- and post-intervention, respectively, as shown in Figure 2.

Single factors Linear Mixed Model for Indoor NO_2_: The presence of an air purifier significantly reduced NO_2_ concentrations (β = −0.45, *p* < 0.001, Table 2), resulting in a 36% decrease from pre-intervention to post-intervention (percent change = 100 × (e^−0.45^ − 1)). Using a linear mixed-effects model, we observed that NO_2_ concentrations were also significantly decreased with an increase in the percentage of time the air purifiers were used in these homes (β = −0.06, *p* < 0.001, Table 2). The percentage of stove usage was found to significantly increase the concentration of NO_2_ (β = 0.07, *p* = 0.003, Table 2), as did the season, with higher levels in winter compared to fall (β = −0.24, *p* = 0.013, Table 2) and summer (β = −0.39, *p* < 0.001, Table 2). The stove type also influenced indoor NO_2_; having a pilot stove increased indoor NO_2_ by 10% (β = 0.74, *p* = 0.004, Table 2). In addition, higher indoor temperatures and humidity levels decreased indoor NO_2_ (β = −0.08, *p* < 0.001, and β = −0.01, *p* = 0.002, respectively; Table 2).

Results from the linear mixed-effects model evaluating fixed effects that predict indoor NO_2_ concentrations and participants as random effects. Estimates represent change in ln NO_2_ (ppb). Air purifier use = The average percent of the time the air purifier was used when placed in the participant’s home. Stove usage is the average percent of time the stove was in use during the sampling period. SE = standard error; n = observations from the four home visits. * Significant *p* value < 0.05.

The presence of an air purifier reduced PM_2.5_ concentrations significantly (β = −0.6, *p* < 0.001, Table 3) with a 45% decrease from pre-intervention to post-intervention (percent change = 100 × (e^−0.6^ − 1)). We also observed that PM_2.5_ concentrations were significantly decreased by every 10% use of the air purifiers in these homes (β = −0.07, *p* < 0.001, Table 3). Unlike NO_2_, the percentage of stove usage did not influence PM_2.5_ concentration (β = 0.03, *p* = 0.229, Table 3). Also, the season did not influence PM_2.5_ concentrations. Other factors that decreased indoor PM_2.5_ were the indoor temperature (β = −0.08, *p* = 0.001), absence of a HEPA vacuum increasing PM_2.5_ (β = 0.36, *p* < 0.001), and frequent use of air fresheners, increasing PM_2.5_ (β = 0.26, *p* = 0.035). These are shown in Table 3.

A multiple-predictor mixed-effects model was employed to examine the relationship between indoor NO_2_ and PM_2.5_ levels, and key predictor variables were determined based on significance in Table 2 and Table 3. The model incorporated fixed effects for air purifier usage, stove usage, and season, while a random effect for household accounted for variability within households. Model selections were based on AIC and BIC values. The final model for NO_2_ demonstrated improved fit with an AIC of 485.9 and BIC of 490.3, while the final model for PM_2.5_ yielded an AIC of 614.2 and BIC of 618.6, both outperforming simpler models. The variables not included in the final multiple LMM were found to be statistically non-significant, and their removal decreased the AIC, indicating a better model fit. We found that the increase of 10% increase in air purifier use decreased NO_2_ concentrations (β = −0.05, *p* < 0.001, Table 4), and 10% increase in stove usage increased NO_2_ (β = 0.06, *p* < 0.001, Table 4), and season, with higher concentrations observed in winter compared to the other seasons (Fall: β = −0.27, *p* = 0.008, Summer: β = −0.45, *p* < 0.001, Table 4). For PM_2.5_, we observed that a 10% increase in purifier use decreased PM_2.5_ concentrations, but stove usage and season did not significantly influence PM_2.5_ (Table 4).

## 4. Discussion

Limited studies have focused on using air purifiers with HEPA/carbon filters to reduce NO_2_; they have focused on simple carbon filters [19,30,41]. Our study demonstrates that air purifiers equipped with HEPA/carbon filters can effectively reduce NO_2_ and PM_2.5_ from gas stoves over an extended period. Our study evaluated the combined effect of air purifier use after four and eight months. We observed that the increase in the percentage of time the air purifier is used resulted in a significant decrease in NO_2_ and PM_2.5_ levels (Table 2 and Table 3). In this study, two air purifiers were placed in the home, specifically in the bedroom and living room, based on findings from earlier studies. Martenies and Batterman (2018) estimated that two air cleaners would remove 84% of outdoor PM_2.5_ in bedrooms when run continuously and remove 77% of the outdoor PM_2.5_ when run 60% of the time [42]. Martenies and Batterman (2018) observed a significant reduction in PM_2.5_ with the use of two air cleaners and attributed this observation to the assumptions that the units had been used continuously (compared to 63–83% in earlier field studies) and that both units had been operated at their maximum speed [42]. Activated carbon has been observed to reduce NO_2_ by absorption [43,44,45]. Matthaios et al. (2023) observed significant reductions by 87% on average (range 80–94.2%) in vehicle NO_2_ levels compared to the on-road concentrations when using activated carbon filters [31]. Reductions in indoor pollutants varied largely by geographical location, air filtration technologies employed, indoor environmental characteristics, and air pollution sources [42,46].

Hansel et al. conducted a randomized clinical trial using air purifiers with HEPA filters, observing a significant reduction in NO_2_ of 24% [47]. Similarly, Paulin et al. found that the placement of air purifiers with HEPA and carbon filters resulted in a 27% decrease in median kitchen NO_2_ concentration after one week of use; at three months post-intervention, the placement of air purifiers resulted in a 19% decrease in median kitchen NO_2_ concentration [19]. Paulin et al. also observed a 23% decrease in median NO_2_ levels after one week of using air purifiers in bedrooms [19]. These findings are similar to our outcomes, where we observed a 36% reduction in indoor NO_2_ with a geometric mean (GSD) of 20.16 (GSD 1.92) ppb at pre-intervention to 12.79 (2.07) ppb post-intervention.

We observed a 45% reduction in PM_2.5_ from 17.12 µg/m^3^ at pre-intervention to 9.16 µg/m^3^ at post-intervention, which is consistent with findings reported by other studies evaluating the use of portable air purifiers in residential settings [21,22,24,41,48,49,50,51]. Our findings align with earlier studies that observed a reduction in PM_2.5_ using HEPA filters. Maestas et al. conducted a randomized, double-blind crossover intervention study where they observed a 60% reduction in indoor PM_2.5_ and a 53% reduction in personal PM_2.5_ exposure [41]. Reiderer et al. found that HEPA purifiers reduced PM_2.5_ by 60% in sleeping areas and by 42% in the living area [40]. Short-term and long-term use of portable air filtration systems has been found to reduce personal and indoor PM_2.5_ exposures [48,52]. Park et al. found that average indoor PM_2.5_ concentrations were reduced by 43% (7.42 to 4.28 μg/m^3^) with the use of an air purifier (*p* = 0.001) [53]. Barkjohn et al. also observed a substantial reduction in indoor PM_2.5_ using air cleaners with HEPA filters, from 34 ± 17 to 10 ± 8 µg/m^3^ [50]. Hansel et al. found a 61% further decrease in indoor PM_2.5_ using air cleaners with HEPA/Carbon filters [47]. This suggests that portable air purifiers, particularly those equipped with HEPA/carbon filters, can effectively reduce indoor PM_2.5_ concentrations, as evidenced by earlier studies and our findings. These findings suggest that the more prolonged use of air purifiers fitted with HEPA/carbon filters has a sustained impact on reducing NO_2_ and PM_2.5_.

In this study, the participants used either a pilot light stove or an autoignition stove. A pilot light is a small flame that continuously burns on a gas stove, while the burners are not in use, which is a source of emissions [17]. We observed that having a pilot light increased indoor NO_2_ in single-predictor models (Table 2). Despite this observation in the single predictor model, we excluded this predictor from the multiple regression model due to the small sample size within the pilot stove category, which could bias the estimates of the regression coefficients and lead to high variance in the estimates. In the simple LMM, higher indoor temperatures and humidity levels are associated with decreased indoor NO_2_ concentrations (Table 2). However, these variables were not included in the final multiple LMM because they were found to be statistically non-significant, and their removal decreased the AIC, indicating a better model fit.

Results from the multiple LMMs, adjusted for stove usage and season, showed that using air purifiers led to a reduction in indoor NO_2_ concentrations. Earlier studies have reported a reduction in NO_2_ with HEPA/Carbon filters; however, this study incorporates stove usage and seasonality to adjust for the effectiveness of this intervention. We observed that stove use increased NO_2_ concentrations (Table 4), which is consistent with other studies that have found gas stoves increase indoor NO_2_ levels [19,54]. We also observed lower indoor NO_2_ concentrations in the fall and summer compared to winter (Table 2 and Table 4). Higher levels of indoor NO_2_ have been observed in the winter months, when indoor heating is more prevalent, and spaces are more enclosed, and lower levels in the summer [11,55,56]. We did not observe any seasonal variation with PM_2.5_ compared to NO_2_. This finding may have occurred due to either sampling methods and indoor activities such as ventilation activities, or it could also be explained that the potential source of PM2.5 may most likely be from outdoor, and we did not conduct any outdoor sampling to determine a filtration index/ratio.

Results from the multiple LMMs indicated that indoor PM_2.5_ concentrations were reduced with HEPA/carbon filter air purifiers, and increased gas stove use did not impact indoor PM_2.5_ concentrations (Table 3 and Table 4). In the simple LMM for PM_2.5_, we observed that PM_2.5_ was increased significantly by the absence of HEPA vacuum in the home (Table 3) and the use of air fresheners 6–7 days a week (Table 3). It is essential to note that after the 8th month of the study period, participants received HEPA vacuum cleaners, indicating that, following the intervention, all homes had HEPA vacuum cleaners, as shown in Table 1. Similar studies have found that using air fresheners contributes to indoor PM_2.5_ concentrations [57,58]. These predictors were excluded from the multiple LMM due to the small sample size within the high air freshener use category. PM_2.5_ arises from various sources other than cooking, such as house dust, smoking, candles, air fresheners, and even outdoor air infiltration from vehicular traffic [59] which explains why we did not observe gas stoves predicting indoor PM_2.5_. We did not observe stove use to influence PM_2.5_; cooking has been observed to peak during cooking, and the time dispersion varied due to indoor activities such as the use of ventilation and opening windows [60,61]. In this study, we average the PM2.5 concentration over the environmental sampling periods (5–7 days) and NO_2_.

The findings from this study are very substantial to ongoing concerns about mitigating indoor air pollution, especially from gas stoves. The reduction of NO_2_ by 36% which shows the effectiveness of the HEPA/carbon filters, is very replicable in other indoor settings. Future studies should consider observing the long-term use of carbon filters, if there is a potential impact on their absorption mechanism. HEPA filters can lower indoor pollutants and lead to improvements in health outcomes or markers of outcomes for different populations [62,63].

### Strengths and Limitations of the Study

This study evaluated the impact of air purifiers fitted with HEPA/carbon filters on NO_2_ and PM_2.5_. Our results show a significant reduction in these pollutants with these filtration units. To our knowledge, this is the first study to demonstrate the effectiveness of air purifiers fitted with HEPA/carbon filters in reducing in-home NO_2_ and PM_2.5_ concentrations in low-income housing. A timer in the air purifier enabled the objective quantification of usage in participants’ homes, rather than relying on subjective data. Additionally, we evaluated the use of the air purifier for 4 to 8 months, indicating that it can provide long-term benefits. Limitations of this study include the absence of a control group. Due to the nature of the research and the population of interest, excluding the potential positive impacts of interventions among older adults would be unethical. Based on the principle of justice, withholding the benefit of the intervention from some of the participants to have a control group will take away from the potential health benefit in this vulnerable population [64]. The findings may have limited generalizability as the study was conducted in Lowell, MA, and only included participants from low-income housing, which may not represent broader populations. Despite not being generalizable to certain age groups and health outcomes, this intervention is adaptable in low-income housing that is limited to refurbishment. We did not determine the indoor air purifier ratio; hence, no estimate on the filtration ratio. We did not collect data on participants’ behaviors related to ventilation habits, such as how often they opened the windows or used an air conditioner, which could influence indoor pollutant levels. Additionally, seasonal variations in air quality were only assessed over one year, which may not capture long-term trends or variations in other geographic regions. No objective measures for fan speed were measured; however, over 50% of the participants used their air purifiers in the medium setting because of the loud noise produced when it was set on high.

## 5. Conclusions

Exposure to NO_2_ and PM_2.5_ indoors can harm health, particularly for older adults who spend a significant amount of time indoors. Therefore, it is crucial to study the impact of HEPA/carbon filter air purifiers on older people’s living environment and health conditions. This study’s findings indicate that air purifiers fitted with HEPA/carbon filters can effectively reduce indoor concentrations of NO_2_ and PM_2.5_. The use of air purifiers for 4–8 months leads to a 36% reduction in indoor NO_2_ and a 45% reduction in PM_2.5_. We observed that stove usage and season influenced indoor NO_2_; increasing the time of air purifier usage also increased NO_2_ and PM_2.5_. Future studies are warranted to enhance our understanding of optimally implementing this intervention in various settings. The findings will enable the tailoring of direct interventions to mitigate these air pollutants, as one-third of US homes cook with gas. Understanding the indoor factors contributing to NO_2_ and PM_2.5_ will help create policies around indoor air quality, gas stove emissions, and nationally recommended levels of these pollutants in the indoor environment, as the current guidelines are primarily directed toward outdoor levels.

## Figures and Tables

**Table 1:** Data Collection and Intervention Schedule.

Timeline	Intervention Period	Environmental Data Collected	Intervention After Data Collection
Visit 1	Pre-intervention	NO_2_, PM_2.5_, temperature, humidity, stove usage, and environmental questionnaire	None (control period months 1–4)
Visit 2	Pre-intervention	NO_2_, PM_2.5_, temperature, humidity, stove usage, and environmental questionnaire	Provided air purifier units with HEPA/carbon/filters in the kitchen and bedroom
Visit 3	Post-intervention	NO_2_, PM_2.5_, temperature, humidity, stove usage, environmental questionnaire, environmental inspection, and air purifier usage	Provide typical multifaceted environmental (HEPA Vacuum and dust mite-proof bed casing) and educational interventions.
Visit 4	Post-intervention	NO_2_, PM_2.5_, temperature, humidity, stove usage, environmental questionnaire, environmental inspection, and air purifier usage	None (final assessment)

**Table 2:** Results from linear mixed models on factors associated with change in indoor ln NO_2_ concentrations in 67 homes.

Independent Variables (Fixed Effects)	n	Estimate	SE	*p*-Value
Presence of air purifier (ref = No) Yes	261	−0.45	0.06	<0.001 *
Air purifier use (per 10% increase in use)	259	−0.06	0.01	<0.001 *
Stove (per 10% increase in use)	257	0.07	0.02	0.003 *
Season (ref = winter)	261			
Fall		−0.24	0.11	0.030 *
Spring		−0.04	0.10	0.704
Summer		−0.39	0.10	<0.001 *
Stove Type (ref Autoignition)	261	0.74	0.26	0.004 *
Average Temperature (°C)	261	−0.08	0.02	<0.001 *
Average Humidity	261	−0.01	0.003	0.001 *
Vent (ref-yes) No	252	−0.07	0.12	0.538
Room type (ref-Wall separates kitchen and living area)	261	0.06	40.11	0.578
Outside Tobacco Smoke (ref-No) Yes	244	−0.02	0.09	0.806
Dryer (ref-No) Yes	244	−0.003	0.13	0.982
Vacuum with HEPA filter (ref-yes) No	245	0.13	0.09	0.127
Trucks (ref-No) Yes	233	−0.17	0.14	0.222
Gas Station (ref-No) Yes	247	0.13	0.17	0.428

Results from the linear mixed-effects model evaluating fixed effects that predict indoor NO_2_ concentrations and participants as random effects. Estimates represent change in ln NO_2_ (ppb). Air purifier use = The average percent of the time the air purifier was used when placed in the participant’s home. Stove usage is the average percent of time the stove was in use during the sampling period. SE = standard error; n = observations from the four home visits. * Significant *p* value < 0.05.

**Table 3:** Results from linear mixed models on factors associated with change in indoor ln PM_2.5_ concentrations in 67 homes.

Independent Variables (Fixed Effects)	n	Estimate	SE	*p*-Value
Presence of air purifier (ref = No) Yes	257	−0.60	0.07	<0.001 *
Air purifier use (per 10% increase in use)	256	−0.07	0.01	<0.001 *
Stove (per 10% increase in use)	253	0.03	0.03	0.229
Season (ref = winter)	257			
Fall		0.01	0.13	0.960
Spring		−0.08	0.12	0.531
Summer		−0.02	0.12	0.901
Stove Type (ref Autoignition)	257	0.21	0.41	0.604
Average Temperature (°C)	257	−0.08	0.02	0.001 *
Average Humidity	257	0.002	0.004	0.663
Vent (ref-yes) No	248	0.04	0.16	0.813
Room type (ref-Wall separates kitchen and living area)	257	0.22	0.16	0.171
Outside Tobacco Smoke (ref-No) Yes	241	0.13	0.12	0.252
Dryer (ref-No) Yes	241	−0.28	0.19	0.193
Vacuum with HEPA filter (ref-yes) No	242	0.36	0.10	<0.001 *
Wall-to-wall carpet (ref-No) Yes	235	0.11	0.18	0.526
Air freshener (ref-never) 6–7 days/week	245	0.26	0.12	0.035 *
Trucks (ref-No) Yes	230	−0.04	0.17	0.823
Gas Station (ref–No) Yes	244	0.39	0.21	0.066

Results from the linear mixed-effects model evaluating fixed effects that predict indoor NO_2_ and PM_2.5_ concentrations and participants as random effects. Estimates represent change in ln PM_2.5_ (µg/m^3^). Air purifier use = The average percent of the time the air purifier was utilized when placed in the participant’s home. Stove usage is the average percent of time the stove was in use during the sampling period. SE = Standard Error. n = observations from the four home visits. * Significant *p* value < 0.05.

**Table 4:** Results from multiple predictor linear mixed models of indoor pollutants in 67 homes.

NO_2_ Predictors (*n* = 255)	Estimate	SE	*p*-Value
Intercept	3.04	0.10	<0.001
Air purifier use (per 10% increase in use)	−0.05	0.01	<0.001 *
Stove (per 10% increase in use)	0.06	0.02	0.005 *
Season			
Fall	−0.27	0.10	0.008 *
Spring	−0.12	0.09	0.193
Summer	−0.45	0.09	<0.001 *
Winter (ref)			
**PM_2.5_ Predictors (*n* = 252)**	**Estimate**	**SE**	***p*** **-value**
Intercept	2.86	0.15	<0.001
Air purifier use (per 10% increase in use)	−0.07	0.01	<0.001 *
Stove (per 10% increase in use)	0.01	0.02	0.576
Season			
Fall	−0.03	0.12	0.795
Spring	−0.18	0.11	0.107
Summer	−0.09	0.11	0.418
Winter (ref)			

Results from the multiple linear mixed-effects model evaluating fixed effects that predict indoor NO_2_ and PM_2.5_ concentrations and participants as random effects. Estimates represent change in ln NO_2_ (ppb) and ln PM_2.5_ (µg/m^3^). Air purifier use = the average percent of the time the air purifier was utilized when placed in the participant’s home. Stove usage is the average percent of time the stove was in use during the sampling period. SE = standard error. n = observations from the four home visits. * Significant *p* value < 0.05.

## Data Availability

The datasets used and/or analyzed during the current study are available from the corresponding author on reasonable request.

## Abbreviations

The following abbreviations are used in this manuscript:

AIC	Akaike Information Criterion
BIC	Bayesian Information Criterion
CHW	Community Health Worker
COPD	Chronic Obstructive Pulmonary Disease
EPA	Environmental Protection Agency
GM	Geometric Mean
GSD	Geometric Standard Deviation
HEPA	High Efficiency Particulate Air
LMM	Linear Mixed Model
NO_2_	Nitrogen Dioxide
PM_2.5_	Particulate Matter 2.5 microns
REDCap	Research Electronic Data Capture
